# Evaluation of muscle-specific and metabolism regulating microRNAs in a chronic swimming rat model

**DOI:** 10.1007/s10974-021-09612-y

**Published:** 2021-12-10

**Authors:** Zsuzsanna Gaál, János Fodor, Attila Oláh, Tamás Radovits, Béla Merkely, János Magyar, László Csernoch

**Affiliations:** 1 Department of Physiology, Faculty of Medicine, University of Debrecen, Nagyerdei blvd. 98, PO Box 22, 4012 Debrecen, Hungary; 2 Heart and Vascular Center, Semmelweis University, Budapest, Hungary; 3 Division of Sport Physiology, University of Debrecen, Debrecen, Hungary

**Keywords:** Skeletal muscle, Epigenetics, microRNA, Metabolism, Physical activity, Prevention

## Abstract

Making benefit from the epigenetic effects of environmental factors such as physical activity may result in a considerable improvement in the prevention of chronic civilization diseases. In our chronic swimming rat model, the expression levels of such microRNAs were characterized, that are involved in skeletal muscle differentiation, hypertrophy and fine-tuning of metabolism, which processes are influenced by chronic endurance training, contributing to the metabolic adaptation of skeletal muscle during physical activity. After chronic swimming, the level of miR-128a increased significantly in EDL muscles, which may influence metabolic adaptation and stress response as well. In SOL, the expression level of miR-15b and miR-451 decreased significantly after chronic swimming, which changes are opposite to their previously described increment in insulin resistant skeletal muscle. MiR-451 also targets PGC-1α mRNA, whiches expression level significantly increased in SOL muscles, resulting in enhanced biogenesis and oxidative capacity of mitochondria. In summary, the microRNA expression changes that were observed during our experiments suggest that chronic swim training contributes to a beneficial metabolic profile of skeletal muscle.

## Introduction

The term epigenetics involves a wide variety of regulatory mechanisms that alter gene expression pattern without changing the sequence of the DNA. Epigenetic alterations are reversible and inheritable, therefore it is both a possibility and a duty for future medicine to restore pathological alterations with the help of targeted epigenetic interventions. Besides DNA-methylation and histone code alterations, non-coding RNAs are also key elements of epigenetic regulation. MicroRNAs are small non-coding RNAs that contain approximately 20–22 nucleotides and regulate posttranscriptionally the expression levels of target genes (Sjögren et al. 2017). In recent years, microRNAs have been proved to be important regulators of myogenesis and skeletal muscle function as well (Sjögren et al. 2017). On the other hand, microRNAs are also key hub points in the regulatory network of metabolic processes under both physiological and pathological conditions (Rottiers and Näär 2012).

As a consequence of their different functions, extensor digitorum longus (EDL) and soleus (SOL) muscles are often used experimentally as representative fast-twitch and slow-twitch muscles, respectively. EDL consists of type IIA and type IIB fibers, while SOL contains mainly type I and type IIA fibers (James et al. 1995; Chemello et al. 2019). Fast twitch fibers of EDL muscles fire more rapidly, and due to their dependence on anaerobic metabolism, they perform well at generating short bursts of strength or speed. However, they become exhausted quickly. In contrast, SOL is a slow-twitch, anti-gravity muscle, that is considered to be constantly active. Fibers of SOL muscles are more efficient at using oxygen to generate adenosine triphosphate (ATP) for continuous, extended muscle contractions. Compared to fast twitch fibers, they fire more slowly, and are able to work over a long time (Chemello et al. 2019; Zierath and Hawley 2004).

Some of the metabolic alterations induced by physical activity in skeletal muscle have already been characterized. Although glycolysis represents a less efficient form of ATP production than oxidative phosphorylation, glycolytic activity is up-regulated in skeletal muscles during increased physical activity. However, lactate accumulation limits the amount of energy that can be produced by glycolysis in compensation for reduced oxidative phosphorylation. Lactate downregulates the glycolytic enzymes hexokinase and phosphofructokinase, furthermore, it also inhibits lipolysis, reducing another potential source of energy (Tang et al. 2012).

Alterations of microRNA expression pattern have also been established in the skeletal muscle samples of patients with metabolic diseases. For example, in skeletal muscle specimens of type 2 diabetes patients, miR-133a and miR-206 were downregulated as compared to normal glucose tolerant people (Massart et al. 2016). Downregulation of miR-206 and miR-133 were reported in skeletal muscle of mice following 12 weeks of high fat diet (Massart et al. 2016).

Based on currently published data, expression levels of microRNAs are strongly influenced by physical activity (Winbanks et al. 2014). Better understanding of the impact of physical training on the expression levels of metabolism-regulating microRNAs could contribute to better prevention of chronic civilization disorders.

The major aim of our experiments, performed on a chronic swimming rat model, was to characterize changes of the expression levels of microRNAs that regulate skeletal muscle function and metabolic pathways. Based on the several targets regulated by distinct microRNAs, some of them are involved in both skeletal muscle differentiation, hypertrophy and fine-tuning of metabolism, which processes are also influenced by chronic endurance training, contributing to the metabolic adaptation of skeletal muscle during physical activity. The microRNA panel that was examined in blood plasma and skeletal muscle specimens of rats exposed to chronic swim training is discussed below.

### MyomiRs: miR-1, miR-133a, miR-206

A set of microRNAs, referred to as myomiRs are expressed both in the heart and skeletal muscle, except for miR-208a, which is cardio-specific, and miR-206, which is a skeletal muscle specific microRNA (Polakovičová et al. 2016). Expression of skeletal muscle myomiRs (miR-1, miR-133a and miR-206) is under the control of myogenic regulatory factors including myogenin, MyoD and Myf5 (Winbanks et al. 2014). MyomiRs target insulin like growth factor 1 (IGF1), regulating protein synthesis and hypertrophy of skeletal muscle (Winbanks et al. 2014). MyomiRs are involved in the metabolic maturation of differentiating skeletal muscle by directing the transition between glycolytic and oxidative metabolic phenotype (Wüst et al. 2018). These microRNAs are also regarded as regulators of exercise adaptation of skeletal muscle (Winbanks 2014).

### Special regulators of cellular homeostasis: mitomiR miR-494 and hypoxamiR miR-210

MiR-494 is so far the most frequently identified mitochondrial microRNA (mitomiR) (Geiger 2017), which regulates mitochondrial biogenesis in skeletal muscle (Yamamoto et al. 2012). MiR-494 negatively regulates the expression of the best characterized mitochondrial sirtuin enzyme, SIRT3 (Geng et al. 2018), which responds dynamically to both exercise and nutritional signals in skeletal muscle to coordinate downstream molecular responses (Palacios et al. 2009). As a direct target of HIF-1α, miR-210 is upregulated by hypoxia, therefore usually referred to as the major hypoxamiR”. Induction of pyruvate dehydrogenase kinase (PDK) by HIF-1α inhibits the enzyme complex of pyruvate dehydrogenase (PDH), blocks the conversion of pyruvate to acetyl coenzyme A (acetyl-CoA), and enhances lactate production (Favaro et al. 2010). The miR-210-mediated repression of iron-sulfur cluster assembly enzyme (ISCU) affects the overall activity of the mitochondrial electron transport chain (Geiger 2017).

### MicroRNAs regulating metabolic pathways (miR-23a, miR-103, miR-107, miR-128a, miR-15b, miR-223, miR-451) and PGC-1α

MiR-23a has a direct impact on glutamine metabolism of cells due to targeting glutaminase enzyme (Rathore et al. 2012). In the steatotic livers of mice fed with a high-fat diet, increased expression of miR-23a was accompanied by an increment of the level of fatty acid synthase enzyme (FASN) (Wang et al. 2017). Endurance training resulted in increased expression level of miR-23a in skeletal muscle (Winbanks et al. 2014), while in muscle obtained from mouse model of chronic kidney disease, decreased expression level of miR-23a was observed (Bhatia and Pattnaik 2016). MiR-107 promotes hepatic lipid accumulation via inhibition of mitochondrial β-oxidation pathway (Vienberg et al. 2017). In accordance with these findings, antagomiR-based silencing of miR-103/107 in mice was followed by decreased liver triglyceride content and improved insulin sensitivity (Kornfeld et al. 2012). According to recently published data, miR-107 has different functional roles in mature adipocytes and in preadipocytes, that can result in either dampened adipogenesis or ectopic fatty acid accumulation (Li et al. 2019). MiR-128a targets insulin receptor substrate 1 (IRS1) in C2C12 myotubes, thereby influencing the activity of PI3K/Akt pathway (Massart et al. 2016). Besides IRS1, insulin receptor (INSR) and further genes of insulin signaling cascades have also been identifed as targets of miR-128a (Dae Ho Lee 2010). Antisense-mediated inhibition of miR-128a in vivo led to skeletal muscle hypertrophy (Winbanks et al. 2014). In addition, miR-128a was found to be upregulated in hibernation and in muscles during torpor (Granata and Jamnick 2018). MiR-15b was upregulated in insulin resistant skeletal muscle (Massart et al. 2016). Due to targeting of insulin receptor by miR-15, the overexpression of this microRNA was confirmed to impair insulin signaling cascade and insulin-stimulated glycogen storage in hepatocytes (Massart et al. 2016). In mammary epithelial cells, inhibition of miR-15b resulted in increased lipid content due to the elevated expression of FASN enzyme (Radovits et al. 2013). Based on recently published data, miR-223 is a critical factor in the maintenance of functional pancreatic ß-cell mass and in the adaptation to metabolic stress as well (Czimmerer et al. 2013). Overexpression of miR-223 increased insulin-stimulated glucose uptake and induced GLUT4 expression in cardiomyocytes (Massart et al. 2016). MiR-223 was also found to inhibit cholesterol biosynthesis and uptake (Aoi et al. 2010). In a mouse model, glycerol kinase mediated hepatic gluconeogenesis was confirmed to be negatively regulated by miR-451 (Bjorkman et al. 2020). AMP-activated protein kinase (AMPK), a key exercise responsive energy sensor described in skeletal muscle, is targeted by miR-451 (Massart et al. 2016). Peroxisome proliferator-activated receptor gamma coactivator 1α (PGC-1α) is phosphorylated by AMPK (Massart et al. 2016), then phosphorylated PGC-1α is deacetylated and activated by sirtuin 1 enzyme (SIRT1) (Nielsen et al. 2010). MiR-451 was found to be upregulated in skeletal muscle in type 2 diabetes (Massart et al. 2016).

Besides microRNAs, expression level of PGC-1α mRNA was also evaluated in the skeletal muscle samples. PGC-1α has key significance in mitochondrial biogenesis within skeletal muscle (Yamamoto et al. 2012). It also promotes a switch from glycolytic to oxidative energy provision. This indicates that modulation of metabolic regulation may improve mitochondrial respiratory function independent of changes in mitochondrial content (Denham and Prestes 2016).

## Materials and methods

### Animals, exercise protocol

All experimental procedures were reviewed and approved by the Ethical Committee of Hungary for Animal Experimentation (Permission Number: PEI/001/2374-4/2015). This investigation conformed to the Guide for the Care and Use of Laboratory Animals provided by the Institute for Laboratory Animal Research (NIH Publication No. 86-23, revised 1996) and to the EU Directive 2010/63/EU. All animals received humane care.

Young adult, age-matched, 57–61 days old male (n = 12) Wistar rats (Toxi-Coop Zrt., Budapest, Hungary) were housed in standard rat cages at a constant room temperature (22 ± 2 °C) and humidity with a 12:12-h light–dark cycle. The animals were allowed access to standard laboratory rat diet and water ad libitum during the whole experimental period.

Exercise training was performed in a water tank divided into six lanes filled with tap water warmed to approximately 30–32 °C at the same time of day in all training sessions as previously described (Nielsen et al. 2014). Exercised rats (n = 6) swam for a total period of 12 weeks, 200 min long session/day and 5 days a week. For adequate adaptation, the duration of first swimming experience was limited to 15 min and increased by 15 min every second training session until 200 min was reached. Control rats (n = 6) were placed into the water for 5 min each day during the 12-week training program.

### Samples

Rats were anesthetized with pentobarbital (60 mg/kg ip). A midline laparotomy was performed, blood samples from the inferior caval vein were collected in tubes pre-rinsed with EDTA, and all animals were euthanized by exsanguination. The blood samples were centrifuged at 3000×*g* for 15 min at 4 °C and stored at − 80 °C. EDL and SOL muscle samples were harvested immediately after sacrifice, snap-frozen in liquid nitrogen and stored at − 80 °C. Due to technical issues, the collection of the above detailed specimens was successful only from 5 chronic swimming rats (n = 5).

Prior to RNA isolation, EDL and SOL skeletal muscle samples were homogenized with Ht Mini Kompakt Homogenizer (Ops Diagnostics, Cat. No.: 1660039) for 4 min, at 300/min frequency.

### RNA isolation

Total RNA fraction was isolated with TRI reagent (MRC, cat. No.: NR118) from 200 µl of blood plasma samples and from homogenized EDL and SOL skeletal muscle specimens. The isolated RNA was dried in a concentrator instrument (Concentrator plus, Eppendorf), resuspended in nuclease-free water (NFW) and stored at − 80 °C temperature. Concentration of the isolated total RNA was evaluated with NanoDrop ND-1000 Spectrophotometer at 260 nm wavelength.

### Reverse transcription (RT) and quantitative polymerase chain reaction (qPCR)

Relative expression levels of 12 different microRNAs (miR-1, miR-133a, miR-206, miR-494, miR-210, miR-23a, miR-103, miR-107, miR-128a, miR-15b, miR-223, miR-451) and PGC-1α mRNA were determined in EDL and SOL skeletal muscle samples. Due to limited amounts, in blood plasma specimens, the relative expression level of only one microRNA (miR-210) could be evaluated.

In case of EDL and SOL samples, 5600–7000 ng of the isolated total RNAs were reverse transcribed into cDNA with Omniscript RT kit (Qiagen, cat. no.: 205113), in 20 µl reaction volumes that contained RNase inhibitor reagent (Promega, N261A). In case of total RNA fractions isolated from blood plasma samples, the reverse transcription of only 700 ng RNA was possible. Length of the RT reaction was 60 min at 37 °C temperature.

The qPCR reactions were performed with SYBR Green Mastermix (Roche, cat. no.: 04887352001), in a Light Cycler 480 Master instrument (Roche) (cat. no. for plates, Roche: 04729692001; cat. no. for sealing foils, Roche: 04729757001). All measurements were conducted in triplicates, 20 µl reaction volume each, containing 5 µl of the previously 2 × diluted RT products. Thermal profile was the following: initiation for 3 min at 95 °C (1×); amplification: 10 s at 95 °C, 30 s at 58 °C, 1 s at 72 °C (45×); cooling: 10 min at 40 °C (1×).

The sequences of primers are listed in Table 1. Based on the sequences of the distinct microRNAs, specific stem-loop structured and forward primers were designed with a freely available online software (http://mirnadesigntool.astridresearch.com), developed by Czimmerer Zs. et al. (Wilfred et al. 2007). A universal reverse primer was also used for all of the microRNA measurements via qPCR. Oligo (dT) primer used for the reverse transcription of PGC-1α mRNA was manufactured by Promega (Cat. No.: C110A). Primers for performing qPCR of PGC-1α cDNA were chosen based on recently published data (Caggiano et al. 2017). The expression levels of all examined genes were normalized to the endogenous sno-202 content of the samples.

**Table 1:** Sequences of primers used for reverse transcription and quantitative polymerase chain reaction

	Stem loop primer for RT	Forward primer for qPCR
miR-1	GTTGGCTCTGGTGCAGGGTCCGAGGTATTCGCACCAGAGCCAAC ATACAC	GTTGGGTGGAATGTAAAGAAGT
miR-133a	GTTGGCTCTGGTGCAGGGTCCGAGGTATTCGCACCAGAGCCAAC CAGCTG	GTGTTTGGTCCCCTTCAAC
miR-206	GTTGGCTCTGGTGCAGGGTCCGAGGTATTCGCACCAGAGCCAAC CCACAC	GTTTGGTGGAATGTAAGGAAGT
miR-494	GTTGGCTCTGGTGCAGGGTCCGAGGTATTCGCACCAGAGCCAAC AGAGGT	GTTTGTGAAACATACACGGGAA
miR-210	GTTGGCTCTGGTGCAGGGTCCGAGGTATTCGCACCAGAGCCAAC CAGTGT	TTGAGCCACTGCCCACAG
miR-23a	GTTGGCTCTGGTGCAGGGTCCGAGGTATTCGCACCAGAGCCAAC GGAAAT	GTTGATCACATTGCCAGGG
miR-103	GTTGGCTCTGGTGCAGGGTCCGAGGTATTCGCACCAGAGCCAAC TCATAG	GTGGAGCAGCATTGTACAG
miR-107	GTTGGCTCTGGTGCAGGGTCCGAGGTATTCGCACCAGAGCCAAC TGATAG	GTAGCAGCATTGTACAGGG
miR-128a	GTTGGCTCTGGTGCAGGGTCCGAGGTATTCGCACCAGAGCCAAC AAAGAG	GTTGTCACAGTGAACCGGT
miR-15b	GTTGGCTCTGGTGCAGGGTCCGAGGTATTCGCACCAGAGCCAAC TGTAAA	GTGTAGCAGCACATCATGG
miR-223	GTTGGCTCTGGTGCAGGGTCCGAGGTATTCGCACCAGAGCCAAC GGGGTA	GTTTGGGTGTCAGTTTGTCAAA
miR-451	GTTGGCTCTGGTGCAGGGTCCGAGGTATTCGCACCAGAGCCAAC AACTCA	GTTTGGAAACCGTTACCATTAC
sno-202	GTTGGCTCTGGTGCAGGGTCCGAGGTATTCGCACCAGAGCCAACCATCA	GTGCTGTACTGACTTGATGAAA
Universal reverse primer for qPCR	GTGCAGGGTCCGAGGT	

PGC1α mRNA	Oligo(dT)15 primer
PGC1α mRNA	Reverese primer for qPCR: CCGTCAGGCATGGAGGAA
PGC1α mRNA	Forward primer for qPCR: CATTTGATGCACTGACAGATGGA

### Evaluation of qPCR data

All qPCR reactions were conducted in triplicates. C_p_ values were determined with the Light Cycler 480 SW 1.5.0 software (Roche). Relative copy numbers were calculated via the ∆C_p_ method. The ratios of the values of the examined and normalization genes gave the relative expression levels.

### Statistical analysis and design of figures

Statistical analysis of the data (Kolmogorov–Smirnov normality test, *t* test, Mann–Whitney Rank Sum Test, Pearson Product Moment Correlation and Spearman Rank Order Correlation) was performed with the SigmaStat 3.0 software. Based on the absolute value of the correlation coefficient (r), the relationship was regarded as very strong (0.7 ≤|r|< 1.00: Pearson, 0.8 ≤|r|< 1.00: Spearman), strong (0.4 ≤|r|< 0.7: Pearson, 0.6 ≤|r|< 0.8: Spearman), moderate (0.3 ≤|r|< 0.4: Pearson, 0.4 ≤|r|< 0.6: Spearman), weak (0.2 ≤|r|< 0.3: Pearson, 0.2 ≤|r|< 0.4: Spearman), or very weak/negligable (0 ≤|r|< 0.2: Pearson, 0 ≤|r|< 0.2: Spearman). Figures were designed with GraphPad Prism 5.00 software. Figures show the relative expression levels of genes, and comparison of the expression of different genes was performed by the comparison of their relative expression levels in all cases.

## Results

### MyomiRs (miR-1, miR-133a, miR-206)

**Fig. 1:**
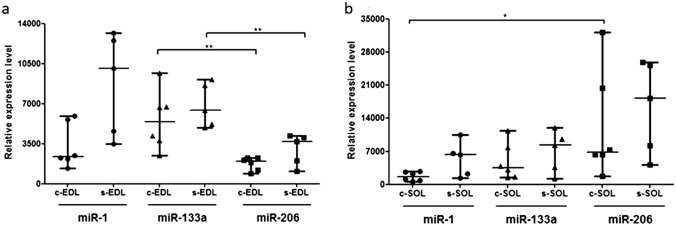
Comparison of myomiR expression levels between EDL and SOL muscles. As a consequence of chronic swimming, increased expression levels of miR-1, miR-133 and miR-206 were observed both in EDL and SOL muscles compared to control animals. **a** Both in control and chronic swimming EDL muscles, the expression level of miR-133a was significantly higher than the expression of miR-206. **b** In control SOL, the expression level of miR-206 was significantly higher than the level of miR-1. Note that the scale for panel *b* is greater than that for (**a)**. *c-EDL* control EDL, *s-EDL* chronic swimming EDL, *c-SOL* control SOL, *s-SOL* chronic swimming SOL. *p < 0.05; **p < 0.01. For statistical analysis, Mann–Whitney and t test were used as it is described in “Materials and methods” section. Samples were obtained from control (n = 6) and chronic swimming rats (n = 5)

Both in EDL and SOL muscles, higher levels of miR-1, miR-133a and miR-206 were measured after chronic swimming, however, these increments were not statistically significant. In control EDL muscles, expression level of miR-133a was higher compared to both miR-1 and miR-206, while after chronic swimming, expression level of miR-1 was the highest among the three examined myomiRs in EDL. Both in control and chronic swimming EDL muscles, the expression level of miR-133a was significantly higher than the expression of miR-206 (Fig. 1a). In case of both control and chronic swimming SOL muscles, the expression level of miR-206 was the highest among myomiRs. In control SOL, the expression level of miR-206 was significantly higher than the level of miR-1 (Fig. 1b). Comparing EDL and SOL muscles, the expression level of miR-206 was significantly higher in SOL than in EDL in control and in chronic swimming muscles as well.

### Special regulators of cellular homeostasis: mitomiR miR-494 and hypoxamiR miR-210

After chronic swimming, the expression level of miR-494 decreased in EDL, and increased in SOL muscles (not significant differences). The expression level of circulating miR-210a in plasma specimens taken after chronic swimming was lower than in the plasma samples of control animals, however, the difference was not statistically significant. After chronic swimming, the expression level of miR-210 was lower in EDL and higher in SOL compared to control EDL and SOL muscles, respectively (not significant differences).

### MicroRNAs regulating metabolic pathways (miR-23a, miR-103, miR-107, miR-128a, miR-15b, miR-223, miR-451) and PGC-1α. miR-23a

**Fig. 2:**
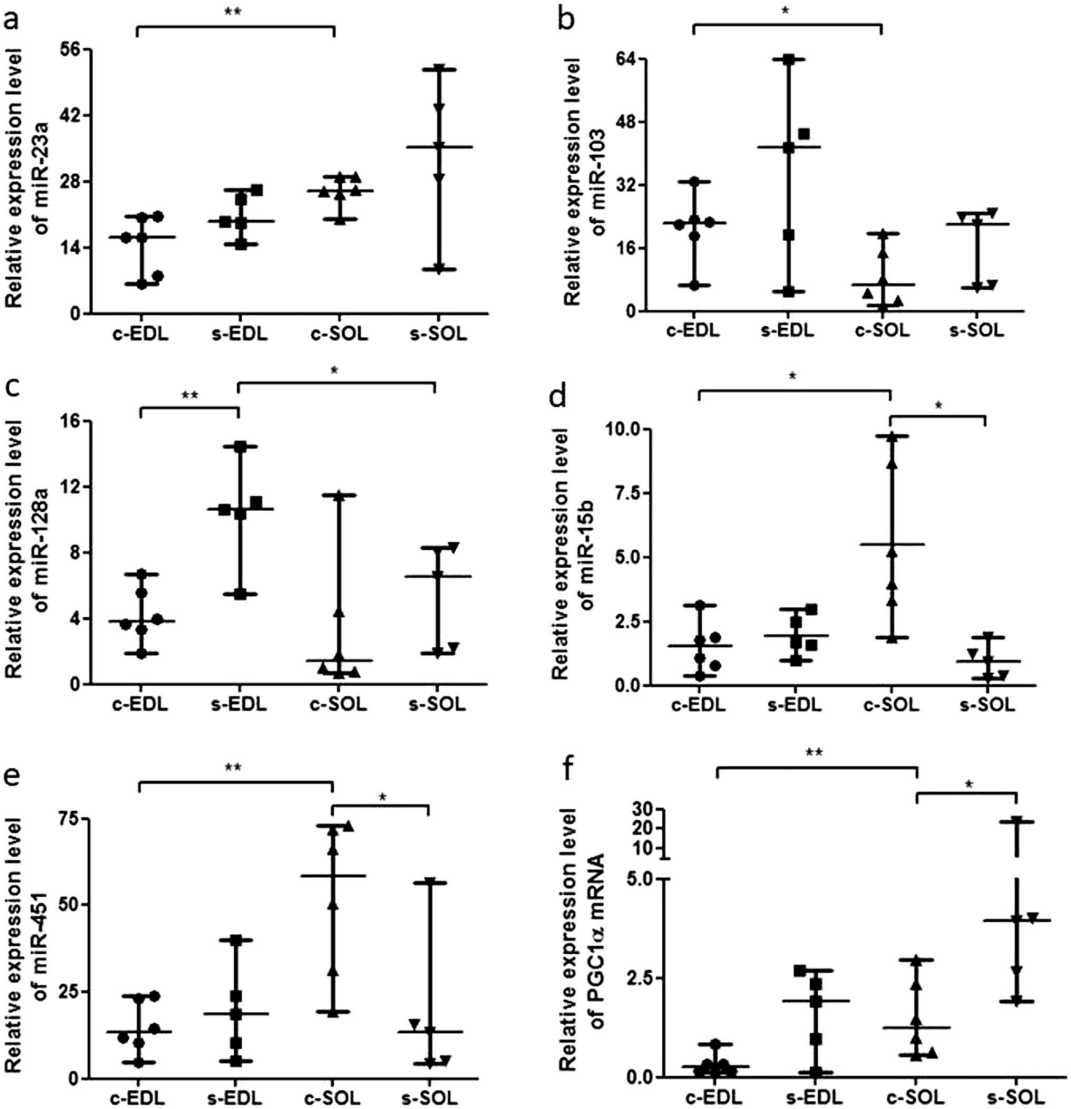
Comparison of metabolism regulating miRNAs and that of PGC1α mRNA between EDL and SOL muscles. **a** The expression level of miR-23a was significantly higher in control SOL than in control EDL muscles. **b** Opposite to miR-23a, the expression level of miR-103 was found to be significantly higher in the control EDL group compared to control SOL samples. **c** In EDL muscles, the expression level of miR-128a was significantly elevated after chronic swimming. Comparing chronic swimming EDL and SOL groups, the expression level of miR-128a was proved to be significantly higher in EDL muscles. **d** The expression level of miR-15b was significantly higher in control SOL than in control EDL muscles. In SOL muscles, expression level of miR-15b was significantly lower after chronic swimming. **e** In control animals, SOL muscles had significantly higher miR-451 content compared to that of EDL. Similarily to miR-15b, significantly decreased expression level of miR-451 was observed after chronic swimming in SOL muscles. **f** The relative expression level of PGC-1α mRNA was significantly higher in control SOL muscles than in control EDL. In SOL muscles, significant increment of PGC-1α mRNA expression level was detected after chronic swimming. *c-EDL* control EDL (n = 6), *s-EDL* chronic swimming EDL (n = 5), *c-SOL* control SOL (n = 6), *s-SOL* chronic swimming SOL (n = 5). *p < 0.05; **p < 0.01. For statistical analysis, Mann–Whitney and t test were used as it is described in “Materials and methods” section

In case of both control and chronic swimming muscles, the expression level of miR-23a was higher in SOL than in EDL muscles, which difference was significant in case of control animals (Fig. 2a). After chronic swimming, the expression level of miR-23a increased in both EDL and SOL muscles (not significant differences). In case of both control and chronic swimming muscles, the expression level of miR-103 was higher in EDL than in SOL muscles, which difference was significant in case of control animals (Fig. 2b). Expression level of miR-107 was higher in control EDL and lower in chronic swimming EDL, compared to control SOL and chronic swimming SOL muscles, respectively. After chronic swimming, higher miR-103 and lower miR-107 levels were detected both in EDL and SOL than in control muscles (not significant differences). In EDL muscle, expression level of miR-128a significantly increased after chronic swimming (Fig. 2c). In SOL muscle, also higher miR-128a level was measured after chronic exercise, but this difference was not statistically significant. Expression level of miR-128a was higher in EDL than in SOL muscle both in control specimens and in the muscle samples obtained from rats after chronic swimming. After swimming, the difference was significant (Fig. 2c). In SOL muscle, expression level of miR-15b significantly decreased after chronic swimming (Fig. 2d). In contrast with SOL muscle, the level of miR-15b increased in EDL muscles in consequence of chronic exercise (not significant difference). Comparing skeletal muscle specimens obtained from control animals, the expression level of miR-15b was significantly higher in SOL (Fig. 2d), while it was higher in EDL muscles after chronic swimming (not significant difference). No statistically significant differences were deteceted between the expression level of miR-223 in EDL and SOL muscles neither in control, nor in chronic swimming animals. After chronic swimming, the level of miR-223 increased in EDL and decreased in SOL muscles compared to control specimens (not significant differences). In control animals, SOL muscles had significantly higher miR-451 content compared to EDL (Fig. 2e), while level of miR-451 was lower in chronic swimming SOL than in chronic swimming EDL muscle. In SOL muscle, expression level of miR-451 significantly decreased after chronic swimming (Fig. 2e). In EDL muscles, level of miR-451 increased after chronic exercise, but this difference was not significant. Expression level of PGC-1α mRNA increased after chronic swimming both in EDL and in SOL muscles. In case of SOL, the difference was significant (Fig. 2f). Comparing EDL and SOL muscles, expression level of PGC-1α mRNA was higher in SOL in both control and chronic swimming animals, which difference was significant in case of control animals (Fig. 2f).

### Correlations between gene expression levels

**Fig. 3:**
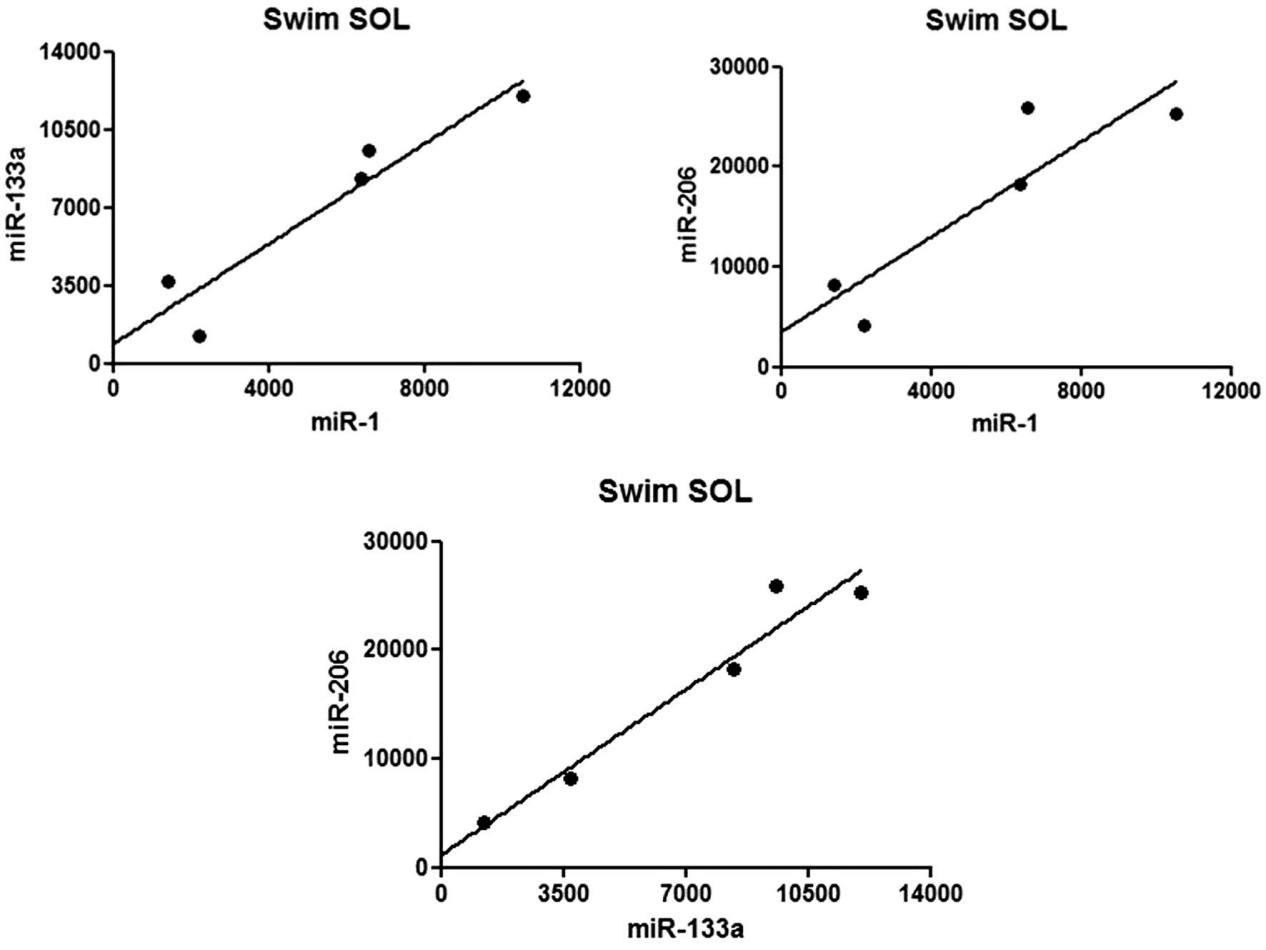
Correlations between the expression levels of myomiRs in chronic swimming SOL muscles. Very strong positive correlation was found in SOL muscles between the expression levels of miR-1 and miR-133a (r = 0.945*), miR-1 and miR-206 (r = 0.892*), furthermore between miR-133a and miR-206 (r = 0.973**). Pearson correlation was performed in all cases. *p < 0.05; **p < 0.01, n = 5

**Fig. 4:**
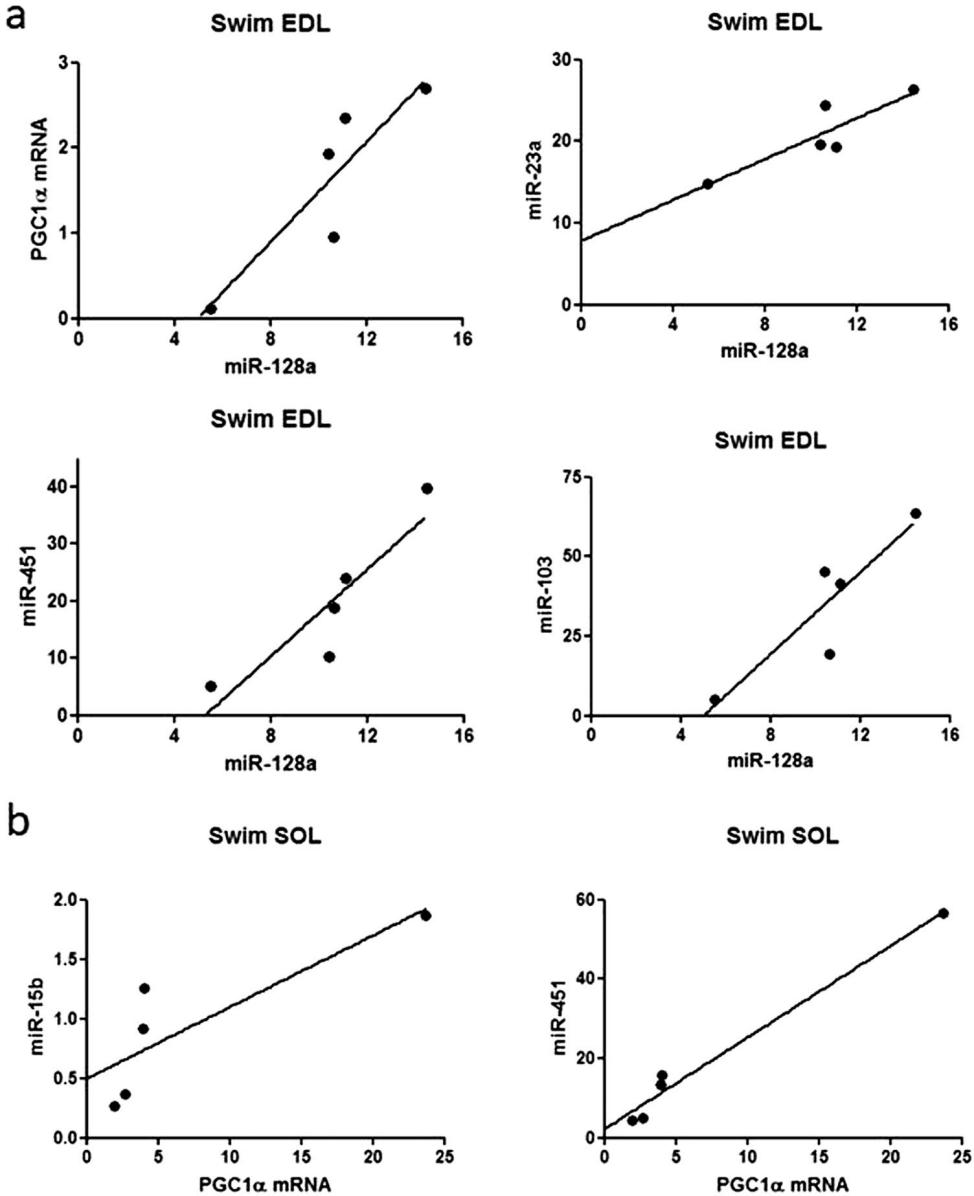
Correlations between the expression levels of metabolism regulating miRNAs and PGC1α mRNA. **a** In EDL muscles following chronic swimming, very strong correlation was found between the expression level of miR-128a and the expression of PGC-1α mRNA (r = 0.893*), miR-23a (r = 0.881*), miR-451 (r = 0.906*) and miR-103 (r = 0.901*). **b** In SOL muscles following chronic swimming, also very strong correlation was observed between the expression level of PGC-1α and the expression of miR-15b (r = 1.00*) and miR-451 (r = 1.00*). Pearson correlation was performed in cases. *p < 0.05, n = 5

**Fig. 5:**
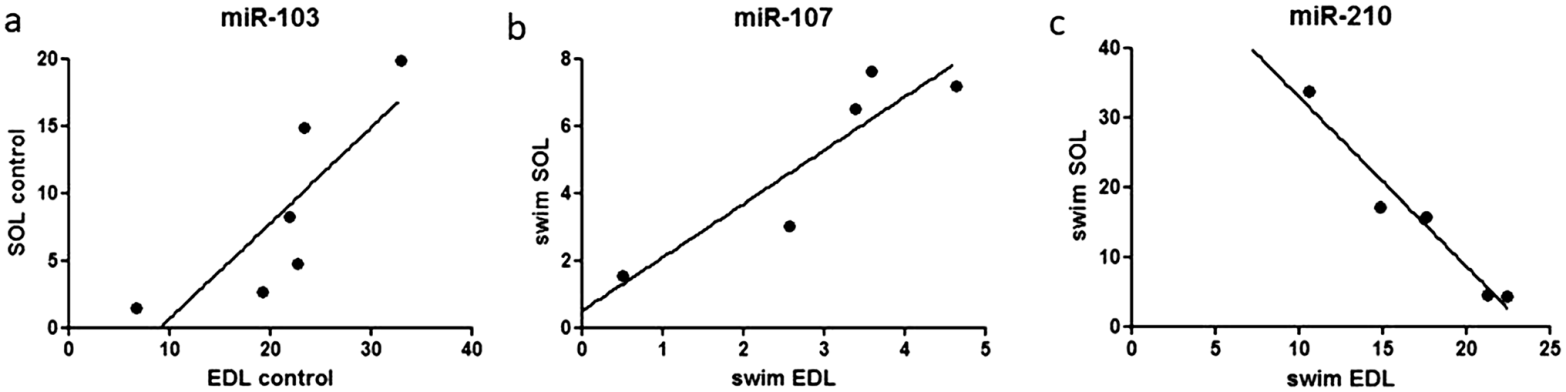
Correlation in the expression levels of miRNAs between EDL and SOL muscles under different conditions. **a** Significant positive correlation of miR-103 expression was found between control EDL and SOL muscles (r = 0.823*, n = 6). **b** Significant positive correlation of miR-107 expression was detected between chronic swimming EDL and SOL muscles (r = 0.904*, n = 5). **c** Significant negative correlation was found between the expression level of miR-210 in chronic swimming EDL and chronic swimming SOL muscles (r = -0.976**, n = 5). Pearson correlation was performed in all cases.*p < 0.05, **p < 0.01

In case of both EDL and SOL muscles, numerous statistically significant correlations were observed between the expression levels of the examined genes (Table 2). In chronic swimming SOL muscles, very strong positive correlation was found between the expression levels of the three examined myomiRs (miR-1, miR-133a, miR-206) (Fig. 3). In chronic swimming EDL muscles, the expression level of miR-128a very strongly correlated with the expression levels of PGC-1α mRNA, miR-23a, miR-451 and miR-103 as well (Fig. 4a). In chronic swimming SOL muscles, the level of PGC-1α mRNA very strongly correlated with the expression levels of miR-15b and miR-451 (Fig. 4b). In case of control animals, the expression level of miR-103 correlated in EDL and SOL muscles, while in chronic swimming animals, the level of miR-107 was confirmed to correlate in EDL and SOL skeletal muscle specimens (Fig. 5a and b, respectively). Significant negative correlation was found between the expression level of miR-210 in chronic swimming EDL and chronic swimming SOL muscles (Fig. 5c), however, expression level of miR-210 in blood plasma samples did not show statistically significant correlation with the level of miR-210 in EDL or SOL muscles.

**Table 2:**
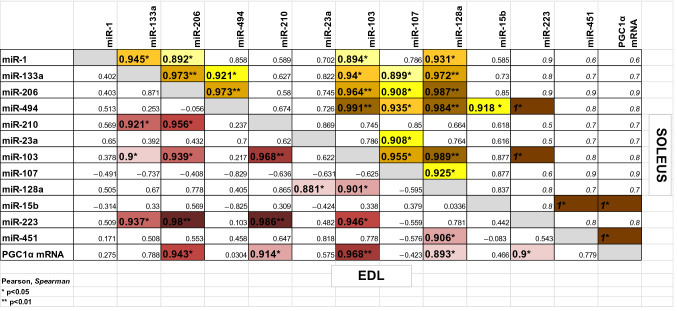
In case of both EDL and SOL muscles, obtained from chronic swimming animals, numerous statistically significant correlations were observed between the expression levels of the examined genes

Pearson or Spearman correlation was performed, italic characters are used in case of Spearman rank correlation

*p < 0.05; **p < 0.01

## Discussion

Physical exercise has a great potential to improve the prevention of chronic civilization diseases. Type 2 diabetes, metabolic syndrome, non-alcoholic fatty liver disease and cardiovascular disorders affect millions of people worldwide, and the abnormal metabolic profile featuring these conditions is under the control of epigenetic regulation in a significant manner. The term epigenetics involves all processes that alter the gene expression pattern without any changes in the sequence of the DNA. Besides DNA methylation and histone modifications, non-coding RNAs including microRNAs regulate all metabolic pathways, and confirmed to be important regulators of skeletal muscle differentiation and metabolic adaptation to physical exercise. Due to their reversibility, microRNA expression level alterations and other epigenetic changes are efficient tools to improve the results of disease prevention.

The major aim of our research was to characterize changes in the expression levels of muscle specific and metabolism-regulating microRNAs in consequence of long-lasting endurance training. In our chronic swimming rat model, the expression levels of skeletal muscle specific myomiRs were evaluated, besides microRNAs that regulate insulin signaling, carbohydrate and fatty acid metabolism, biogenesis and redox-dependent pathways of mitochondria. These measurements were completed with the assessment of PGC-1α mRNA level in EDL and SOL muscles. Up to our knowledge, no data has been previously published about the expression of these genes in a chronic swimming rat model.

In both control and chronic swimming SOL specimens, the expression level of miR-206 was the highest among the three examined myomiRs, and the expression level of miR-206 was significantly higher in control and swimming SOL muscles compared to control and swimming EDL, respectively. These observations are parallel with the recently published results according to which miR-206 is enriched in slow muscles (Zhao et al. 2019). In our model, the expression level of miR-1 increased in both EDL and SOL muscles after chronic swim training, however, the difference was not statistically significant. In human studies, level of miR-1 increased after short exercise, and decreased after long-lasting endurance training (Menshikova et al. 2006). However, in endurance athletes, miR-1 was found to be upregulated, and it correlated positively with VO_2max_ value as well (Li et al. 2021). These controversial findings may result from differences between species and exercise protocols too.

In slow and fast muscles, opposite changes of miR-210 expression level were observed, and a significant negative correlation was confirmed between miR-210 expression levels of chronic swimming EDL and chronic swimming SOL muscles. Due to the several regulatory factors and target genes of microRNAs, tissue microenvironment has great influence on the primary function of a distinct microRNA, which may explain the antiparallel expression level changes of miR-210 in EDL and SOL muscles after chronic swim training. Similarily to miR-210, opposite changes of expression levels were found in case of both miR-223 and miR-494 in EDL and SOL muscles after chronic endurance training.

In accordance with previously reported findings (Winbanks et al. 2014), increased expression level of miR-23a was observed in our chronic swimming rat model, however, this increment was not statistically significant. In a mouse model of chronic kidney disease (CKD), low expression level of miR-23a was deteceted in skeletal muscle samples, and it is suspected, that microRNA expression changes caused by endurance training may have protective role against muscle atrophy in CKD (Rathore et al. 2012).

In response to 12 weeks endurance training, the levels of circulating miR-103 and miR-107 were previously found to be upregulated (Ahonen et al. 2019). In our chronic swimming rat model, increased expression level of miR-103 was observed, but the level of miR-107 decreased, however, these changes were not statistically significant. Both genes are encoded within the introns of pantothenate kinase (PANK) gene and regarded as central regulators of cellular acetyl-CoA levels (Chakraborty et al. 2014), but on the other hand, their expression levels may be differentially regulated by currently unknown mechanisms in skeletal muscle leading to metabolic alterations that should be further characterized.

The expression level of miR-128a was significantly higher in EDL muscles after chronic swimming. MiR-128a is considered to be a “redoximiR”, that is implicated in stress responses of skeletal muscle by inhibiting redox-dependent pathways induced by oxidative stress (Chu et al. 2018). Therefore, our results suggest that besides metabolic adaptation, regular swimming until exhaustion also leads to stress adaptation in skeletal muscle. Altogether, the “redoximiR” function of miR-128a seems to be more emphatic in the primarily glycolysis-dependent EDL type of skeletal muscle.

In SOL muscles, the expression level of miR-15b significantly decreased after chronic swimming, which is opposite to the changes that were previously reported in insulin-resistant skeletal muscle (Massart et al. 2016). Besides its implication in insulin signaling, miR-15b also inhibits the expression of SETD3 histone methyltransferase enzyme, therefore regulating muscle cell differentiation (Vickers et al. 2014). In contrast to our findings in SOL muscles, the expression level of miR-15b was higher in swimming EDL muscles compared to control EDLs. We suggest that primary function of miR-15b may be different in slow and fast muscles, that have to be further characterized.

The expression level of miR-451 significantly decreased in SOL muscles after chronic swimming. Since AMPK is a direct target of miR-451, decreased expression of miR-451 leads to increased activation of PGC-1α by AMPK, resulting in enhanced mitochondria biogenesis (Massart et al. 2016). In type 2 diabetes, increased miR-451 levels were reported (Massart et al. 2016). Therefore, decreased expression of miR-451 may contribute to improved metabolic landscape of skeletal muscle.

In our chronic swimming rat model, a statistically significant increment of the expression level of PGC-1α mRNA was observed in SOL muscles. Therefore we suggest, that chronic swim training results in higher mitochondria content of skeletal muscle. Elevated PGC-1α mRNA expression may exert a beneficial effect on metabolic profile as well, since reduced capacity for oxidative metabolism contributes to both age-associated insulin resistance and fat accumulation within skeletal muscle (Zhuo et al. 2016).

In summary, in our chronic swimming rat model, some novel significant changes of microRNA expression levels were described. The expression level of PGC-1α mRNA was also evaluated in fast and slow types of skeletal muscle. In addition, significant correlations were revealed between the expression levels of microRNAs that are involved in differentiation and metabolic pathways of muscle cells. Resulting from the individually varying fiber composition of EDL and SOL muscles, large variance in data sets occured in some cases. Besides the low number of cases, one of the major limitations of our study is the lack of the measurement of exact metabolic paramteres such as glucose uptake and oxygen consumption. Due to the limited amount of samples, the detailed evaluation of microRNA targets could not be performed. PGC1α was chosen based on its significance in mitochondrial biogenesis and metabolic adaptation of skeletal muscle.

All in all, our observations suggest that chronic endurance training results in microRNA expression level changes that contribute to a beneficial metabolic profile and enhanced mitochondria content of skeletal muscle. Better understanding of how different kinds of physical activities influence the expression levels of such metabolism-regulating microRNAs that are involved in the pathogenesis of chronic civilization diseases can help us to provide risk-adapted, personalized consultancy for both patients and healthy individuals, improving life expectancy and quality of life as well.

## Data Availability

The datasets analysed during the current study are not publicly available in accordance with the General Data Protection Regulation, but data sets may be available from the corresponding author on reasonable request.

## Abbreviations

Acetyl-CoA: Acetyl coenzyme A
AMPK: AMP-activated protein kinase
ATP: Adenosine triphosphate
CKD: Chronic kidney disease
EDL: Extensor digitorum longus
FAS: Fatty acid synthase enzyme
IGF1: Insulin like growth factor 1
INSR: Insulin receptor
IRS1: Insulin receptor substrate 1
ISCU: Iron-sulfur cluster assembly enzyme
NFW: Nuclease-free water
PANK: Pantothenate kinase
PDH: Pyruvate dehydrogenase
PDK: Pyruvate dehydrogenase kinase
PGC-1α: Peroxisome proliferator-activated receptor gamma coactivator 1α
qPCR: Quantitative polymerase chain reaction
RT: Reverse transcription
SIRT1: Sirtuin 1 enzyme
SOL: Soleus
